# Supplementary material for: The Concordance of Secondary Pathogenic Germline Variants Identified by Tumor Genomic Profiling in Adult Solid Tumor Patients at Two US Community Cancer Centers
Source: Genes (Basel). 2025 Dec 9;16(12):1476. doi: 10.3390/genes16121476 (PMC12732780; doi:10.3390/genes16121476)

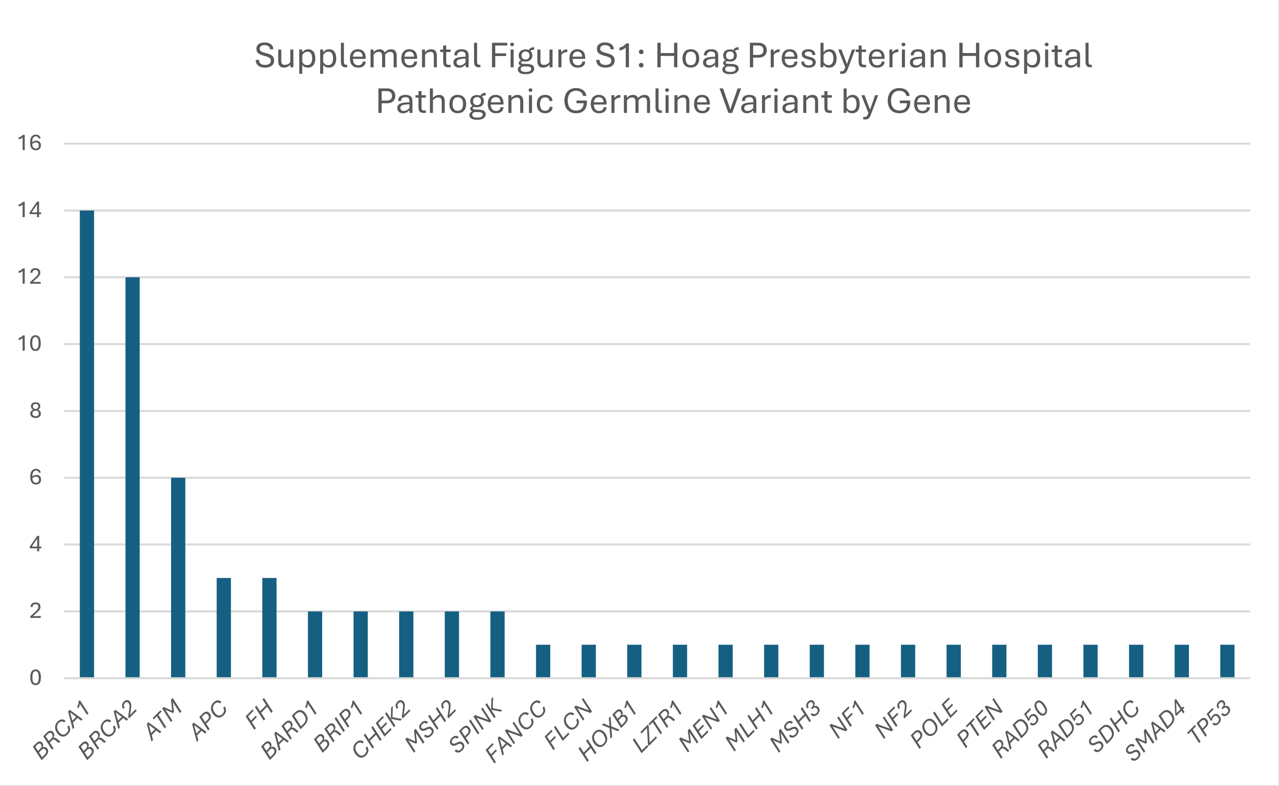
Supplemental Figure S1: Hoag Presbyterian Hospital Pathogenic Germline Variant by Gene
16
14
12
10
8
6
4
2
0
BRCA1
BRCA2
ATM
APC
FH
BARD1
BRIP1
CHEK2
MSH2
SPINK
FANCC
FLCN
HOXB1
LZTR1
MEN1
MLH1
MSH3
NF1
NF2
POLE
PTEN
RAD50
RAD51
SDHC
SMAD4
TP53

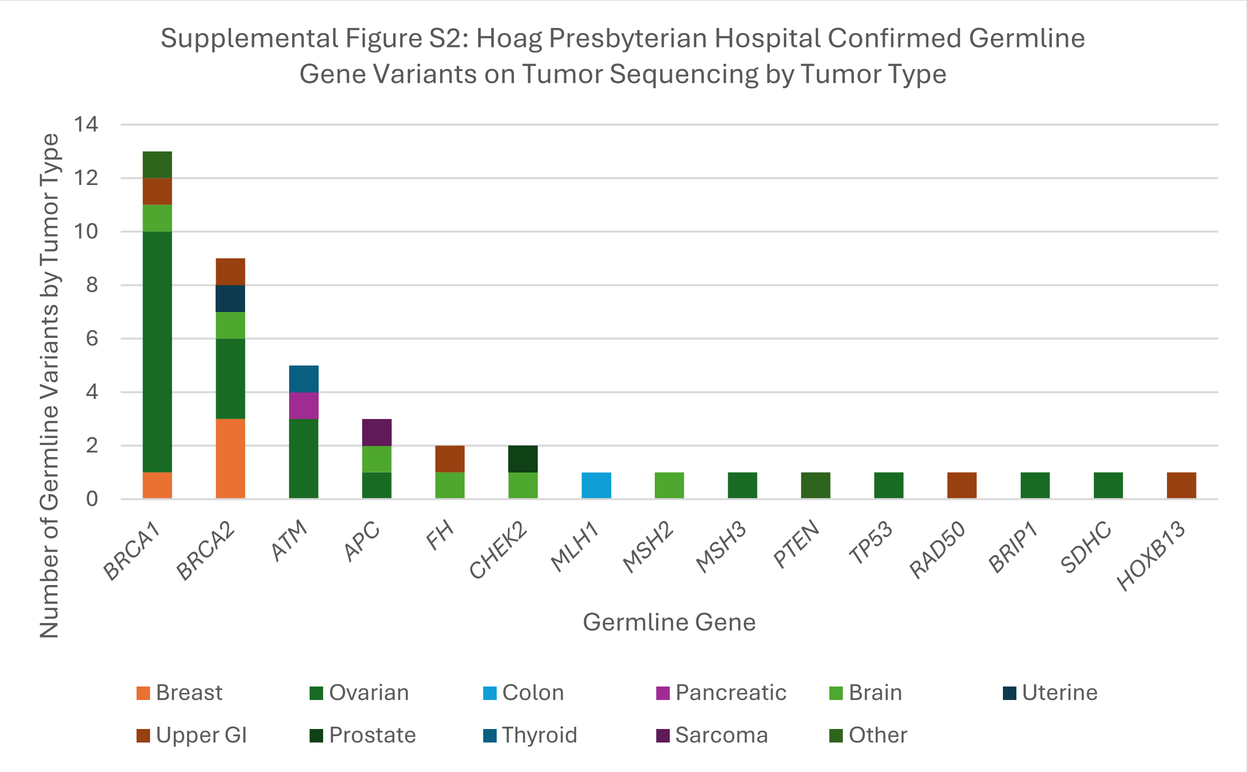
Supplemental Figure S2: Hoag Presbyterian Hospital Confirmed Germline Gene Variants on Tumor Sequencing by Tumor Type
Number of Germline Variants by Tumor Type
14
12
10
8
6
4
2
0
BRCA1
BRCA2
ATM
APC
FH
CHEK2
MLH1
MSH2
MSH3
PTEN
TP53
RAD50
BRIP1
SDHC
HOXB13
Germline Gene
Breast
Ovarian
Colon
Pancreatic
Brain
Uterine
Upper GI
Prostate
Thyroid
Sarcoma
Other

Supplemental Figure S3: Hoag Presbyterian Hospital Number of Patients by Tumor Type

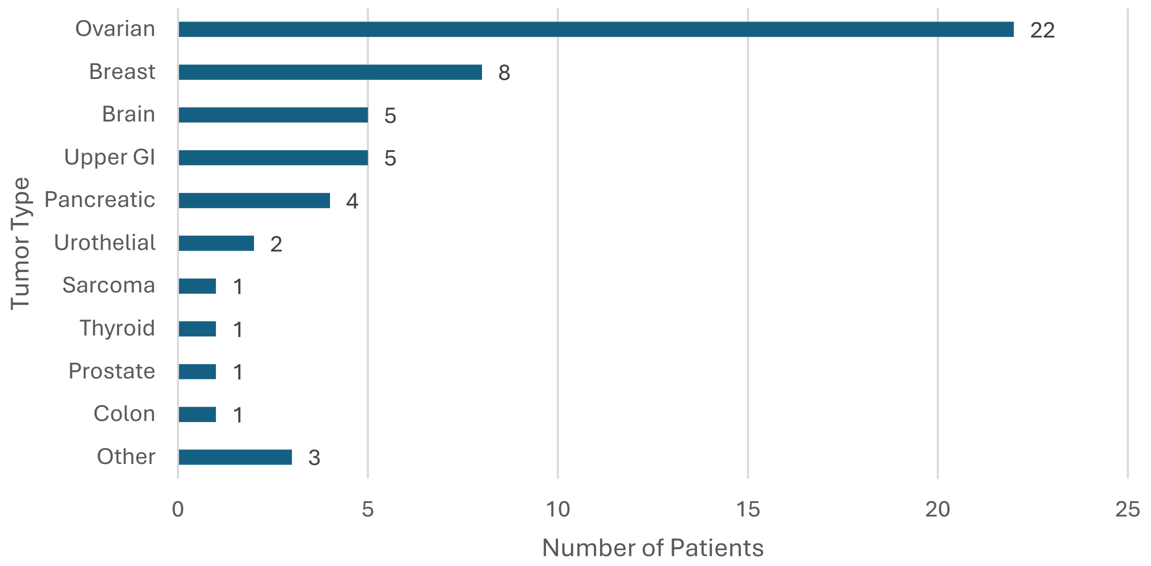

Supplemental Figure S4: Hoag Presbyterian Hospital Variant Allele Frequencies of Confirmed Pathogenic Germline Variants

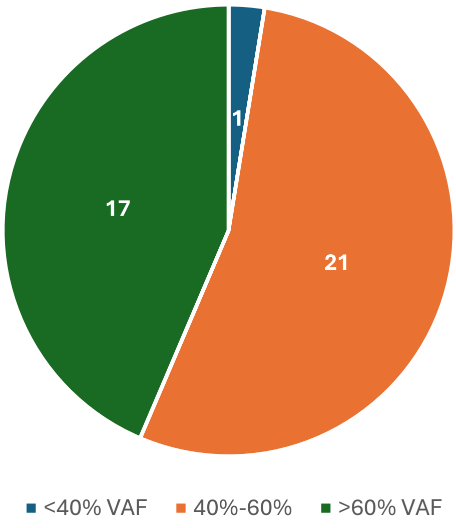

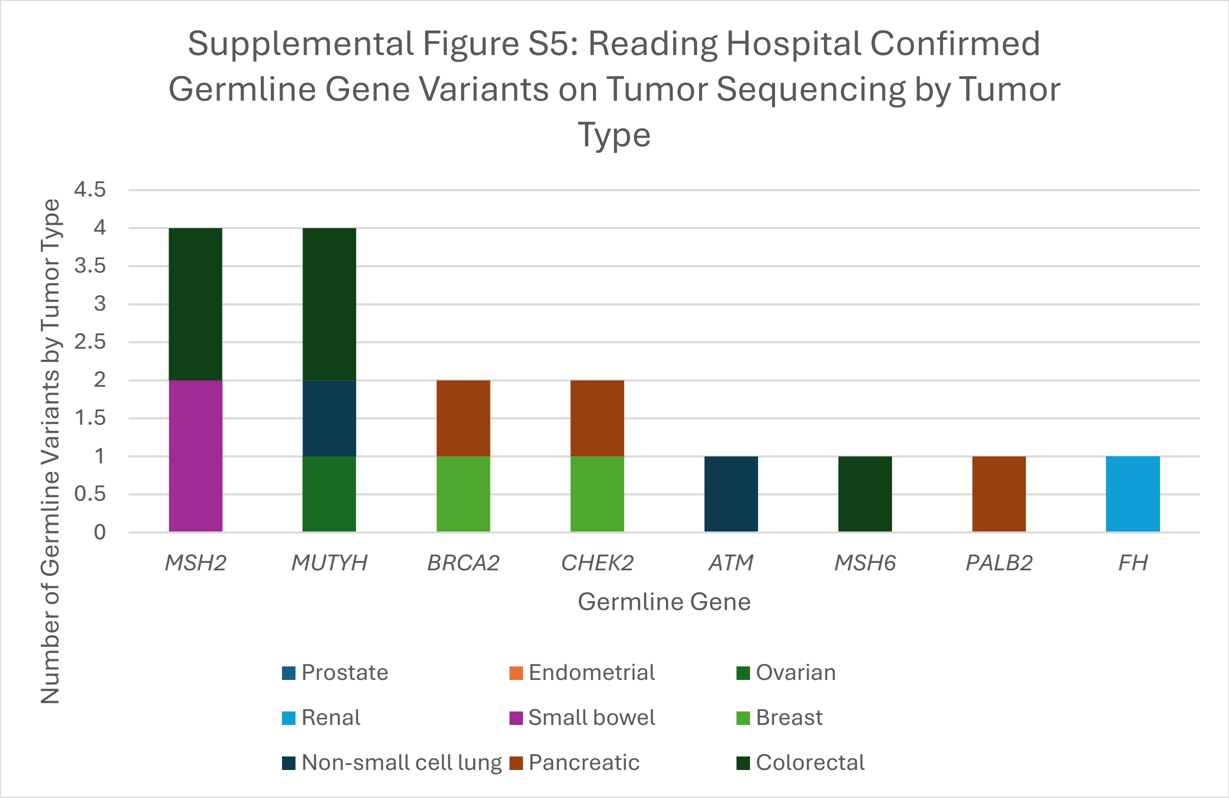

Supplemental Figure S5: Reading Hospital Confirmed Germline Gene Variants on Tumor Sequencing by Tumor Type
Number of Germline Variants by Tumor Type
4.5
4
3.5
3
2.5
2
1.5
1
0.5
0
MSH2
MUTYH
BRCA2
CHEK2
ATM
MSH6
PALB2
FH
Germline Gene
Prostate
Endometrial
Ovarian
Renal
Small bowel
Breast
Non-small cell lung
Pancreatic
Colorectal

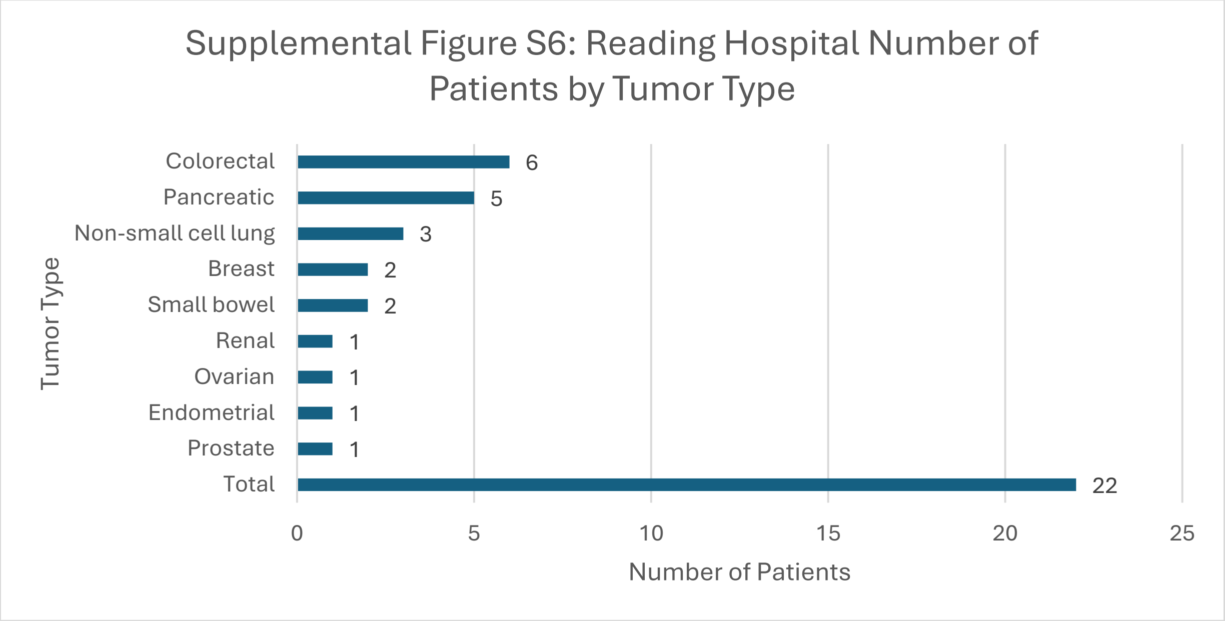

Supplemental Figure S6: Reading Hospital Number of Patients by Tumor Type
Tumor Type
Colorectal
6
Pancreatic
5
Non-small cell lung
3
Breast
2
Small bowel
2
Renal
1
Ovarian
1
Endometrial
1
Prostate
1
Total
22
0
5
10
15
20
25
Number of Patients

Supplemental Figure S7: Reading Hospital Variant Allele Frequencies of Confirmed Pathogenic Germline Variants

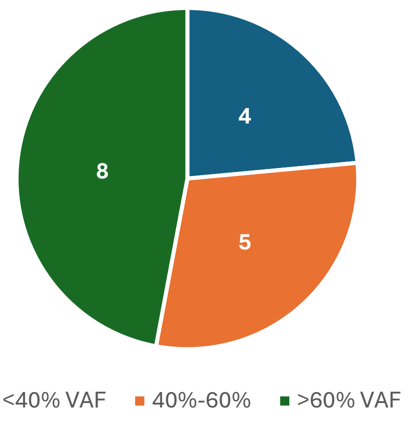

Supplement: Supplementary file 1 [file genes-16-01476-s001.zip › genes-3929057-supplementary.pdf]
